# Gut microbiota were altered with platelet count and red blood cell count in immune thrombocytopenia patients with different treatments

**DOI:** 10.3389/fcimb.2023.1168756

**Published:** 2023-05-15

**Authors:** Xue Rui, Yanjun Fu, Jie Cai, Yu Zhang, Qiang Fu, Chengtao He

**Affiliations:** ^1^ Red Cell Reference Laboratory, Nanjing Red Cross Blood Center, Nanjing, Jiangsu, China; ^2^ Department of Microbiology, The First Affiliated Hospital of Jiamusi University, Jiamusi, Heilongjiang, China; ^3^ Department of Blood Management, Administrative Office, Nanjing Red Cross Blood Center, Nanjing, China

**Keywords:** immune thrombocytopenia (ITP), 16s rRNA sequencing, gut microbiota, biomarker, platelet, red blood cell

## Abstract

The gut microbiome is clearly linked to the development of various autoimmune diseases, however, its association with immune thrombocytopenia (ITP) is less well understood. The current study collected 73 samples, including 36 from healthy individuals and 37 from ITP patients. The gut microbial community was assessed using 16s rRNA sequencing. Findings illustrated that the abundance of key microbiota was significantly higher in the ITP group. This group was further divided into three subgroups that received different treatments for ITP. A random forest model was used to predict the key microbiota and the identified bacteria were shown to easily distinguish between the healthy and the ITP treatment groups. Microbial function annotation and difference analysis showed that drug treatment changed the gut microbiota and may play a role in inducing host autoimmune responses by changing microbial metabolism pathways. Clinical indices also correlated negatively with changes in the microbiota after treatment. In summary, ITP patients who received drug treatment had significant differences in their microbiota along with a high abundance of bacteria. Thus, the microbiome could be used as a biomarker to distinguish between healthy and ITB groups. The key differential bacteria could help to regulate the number of platelets in ITP patients and provide a red blood cell overstock.

## 1 Introduction

Immune thrombocytopenia (ITP), also known as idiopathic thrombocytopenic purpura, is an autoimmune disease that is mainly characterized by isolated thrombocytopenia (Cooper and Ghanima, 2019; Provan et al., 2010; Provan et al., 2019). This disease is heterogeneous, ranging from slight bleeding of the skin and mucosal membranes to life-threatening bleeds (Cooper and Ghanima, 2019). ITP affects about 45–105 cases/million adults, and about 46 cases/million children (Zhang et al., 2020). The pathogenesis and etiology of this condition remain poorly understood but it is generally believed that antibody-wrapped platelets interact with Fcγ receptors and are destroyed in the spleen and liver, eventually leading to ITP (Zufferey et al., 2017; Cooper and Ghanima, 2019). Genetic and environmental factors may play an important role in disease development. Genetic factors associated with ITP pathogenesis include HLA FcγR and some inflammation-related gene mutations (Nomura et al., 1998; Breunis et al., 2008; Satoh et al., 2013; Li et al., 2017), while environmental factors include viruses and other factors that induce infection, including hepatitis C virus core envelope 1 protein or human immunodeficiency virus GP120, which interact with platelet glycoprotein to trigger autoimmune responses (Bettaieb et al., 1992; Zhang et al., 2009). While FcγRIIb expression on monocytes is inhibited in patients with *Helicobacter pylori* infection (Kodama et al., 2007), platelet and cytokine levels return to normal after the infection is controlled. Other environmental factors that induce infection may trigger ITP through similar mechanisms.

At least 1,000 kinds of microbiota can be found in the human intestinal tract, accounting for a huge gene library that encodes 100 times as many genes as are found in the human genome. These microbiota are complex and interconnected to maintain host health (Hiseni et al., 2021). Homeostasis of the gut microbiota plays a vital role in ensuring host metabolism and immunity and disordered intestinal microbiota are associated with many diseases, such as type II diabetes, atherosclerotic cardio-cerebrovascular disease, inflammatory bowel disease, rheumatoid immunity, and compulsory spondylitis (Qin et al., 2012; Jie et al., 2017; Collison, 2019; Franzosa et al., 2019). Studies have shown that supplementing probiotics or fecal microbiota transplantation (FMT) can be clinically beneficial to patients with autoimmune diseases (Borody et al., 2011). Thus, it is reasonable to assume that ITP patients could respond positively to FMT.

Few studies have assessed the intestinal microbiome of ITP patients or investigated changes that occur after drug treatment. The current study collected the feces of ITP patients receiving different treatments, and used 16s rRNA sequencing to explore differences in their gut microbiota and assess the relationship between the intestinal microbiome and various clinical indices.

## 2 Materials and methods

### 2.1 Sample collection

Stool samples were obtained from 73 individuals seen at the First Affiliated Hospital of Jiamusi University between April 2020 and February 2021. Of these, 36 samples were from healthy volunteers and 37 were from ITP patients. Samples were only obtained from subjects who did not take antibiotics, hormones, kaiseki, or any other drugs that affect intestinal microorganisms for 6 months prior to sampling. Individuals who were pregnant or lactating or had digestive and cardiovascular diseases, diabetes, metabolic syndrome, intestinal surgery, irritable bowel syndrome, and autoimmune diseases were excluded from the study. Samples were only collected from firm stools. All included subjects signed the informed consent form before sample collection and passed the Ethics Committee of Nanjing Red Cross Blood Center (2023-001-01) examination. Patient information was recorded at the same time as stool collection (**Table 1**). To ensure the accuracy of the experiment, the platelet counts were manually counted by Microscope DMi1 400 x (Leica, Germany) for these ITP patients with low platelets, and the RBC, Hemoglobin, Hematocrit, and Thrombocytocrit were measured by Automated Hematology Analyzer XN 3000 (Sysmex Corporation, Japan). The normal range for platelet counts were 100-300 × 10^9^/L, red blood cell counts were 3.8-5.1 × 10^12^/L, hemoglobin was 115-150 g/L, hematocrit was 0.35-0.45 L/L and hematocrit was 0.19-0.36.

**Table 1:** Demographic characteristics of the disease groups.

	Ami_Leu	Eltro	Leukin11	Overall
	(N=11)	(N=15)	(N=11)	(N=37)
AGE				
Mean (SD)	67.4 (6.92)	73.2 (10.2)	67.1 (6.96)	69.6 (8.70)
Median [Min, Max]	67.0 [57.0, 81.0]	77.0 [55.0, 88.0]	69.0 [53.0, 76.0]	69.0 [53.0, 88.0]
Gender				
Mean (SD)	0.364 (0.505)	0.400 (0.507)	0.273 (0.467)	0.351 (0.484)
Median [Min, Max]	0 [0, 1.00]	0 [0, 1.00]	0 [0, 1.00]	0 [0, 1.00]
RBC				
Mean (SD)	3.71 (0.547)	3.12 (0.843)	3.66 (0.474)	3.46 (0.708)
Median [Min, Max]	3.78 [2.95, 4.62]	3.07 [1.43, 4.44]	3.63 [2.81, 4.25]	3.60 [1.43, 4.62]
Pla				
Mean (SD)	26.2 (9.37)	20.3 (7.44)	30.0 (7.77)	24.9 (8.95)
Median [Min, Max]	26.0 [15.0, 48.0]	21.0 [2.00, 32.0]	31.0 [18.0, 45.0]	23.0 [2.00, 48.0]
Hmb				
Mean (SD)	113 (16.6)	97.4 (29.1)	110 (11.6)	106 (22.2)
Median [Min, Max]	117 [89.0, 141]	105 [27.0, 138]	110 [87.0, 124]	110 [27.0, 141]
Hem				
Mean (SD)	0.357 (0.0463)	0.315 (0.0641)	0.359 (0.0466)	0.340 (0.0570)
Median [Min, Max]	0.368 [0.281, 0.427]	0.325 [0.227, 0.409]	0.358 [0.298, 0.456]	0.357 [0.227, 0.456]
PCT				
Mean (SD)	0.0964 (0.0731)	0.0453 (0.0247)	0.109 (0.0493)	0.0795 (0.0569)
Median [Min, Max]	0.0800 [0.0300, 0.300]	0.0400 [0.0100, 0.0900]	0.100 [0.0400, 0.200]	0.0800 [0.0100, 0.300]

### 2.2 DNA extraction, 16s rRNA library preparation, and sequencing

Zymo Research BIOMICS DNA Microprep Kit (Cat # D4301) was used to purify gDNA, agarose electrophoresis (0.8%) was used to check gDNA integrity, and Tecan F200 was used to measure the nucleic acid concentration (PicoGreen dye method). The primers, 5’- 3’: 515F(5’- GTGYCAGCMGCGCGGTAA-3’) and 806R(5’- GGACTACHVGGGTWTCTAAT-3’), were used to amplify the V4 region of the 16s bacterial rRNA sequence and the Zymoclean Gel Recovery Kit (Cat # D4008) was used to recycle and purify the amplified product. The Qubit2.0 Fluorometer (Thermo Scientific) was used to determine the fragment concentration, and the NEBNext Ultra II DNA Library Prep Kit from Illumina (NEB#E7645L) was used to prepare the library. The libraries that passed quality control (QC) were loaded onto a Illumina sequencer to generate sequencing reads using the pair end 250 base pair sequencing strategy.

### 2.3 Quality control

QIIME2 software (Bolyen et al., 2019) was used to separate the sequences of each sample from rawdata, remove the barcode sequence, and conduct quality QC. Sequences with an average quality of <25, length of <200 bp, or more than two fuzzy bases (N) were removed. The Uchime algorithm (Edgar et al., 2011) and gold database (v6 version: https://ngdc.cncb.ac.cn/databasecommons/database/id/504) were then used to remove mosaics and obtain data efficient tags.

### 2.4 Microbiome composition and functional annotation

Usearch software (10.0.240 version: http://drive.com/uparse/) was used to cluster OTUs at the 97% consistency level, and the sequence with the highest frequency was selected as the representative OTU. UCLUST taxonomy and the SILVA database (132 version: https://www.arb-silva.de/) were used for annotation analysis. PyNAST was used to perform multiple alignment of representative sequences, and FastTree was used to build an evolution tree. Sample data were homogenized, and the sample with the least amount of data served as the baseline. The 16S rRNA gene sequencing data were clustered and annotated in the SILVA database and the pre-calculated correlation matrix was used to obtain the microbial classification spectrum based on the KEGG database. The results were then corrected according to the copy number of the 16S rRNA gene in the NCBI database. Functional prediction was determined using the functional microorganism gene spectrum in the KEGG database with linear prediction.

### 2.5 Alpha and beta diversity analyses

Vega Packages (Oksanen et al., 2019) were used to calculated alpha diversity and a boxplot was created to show the results. Bray-Curtis distances were used to calculate the distances between different samples and a PCoA diagram was created to show the beta diversity results.

### 2.6 Differential and random forest analyses

The EasyMircoPlot package (Liu et al., 2021) were used to filter core bacteria. The mini_ relative=0.001 and mini_ Ratio=0.5, tax_Plot modules were used to calculate differential microbiota. The random forest model was used to assist in screening and assess the differences between key bacteria. A total of 10 replicates were used in the random forest model and the ROC was used to evaluate the results. Stamp software was used to analyze the functional difference.

### 2.7 Analysis of the microbial co-occurrence network

The Igrap package in R (www.r-project.org) was used to calculate the correlation coefficient between the nodes of the co-occurrence network using data from the core microorganisms. A heat map of the co-occurrence network and node importance was drawn using the igrap package in R.

### 2.8 Statistical analysis and visualization

All statistical analyses were conducted using R software (version 4.2.2). A one-way analysis of variance (ANOVA) and LSD were used for multiple groups in the difference analysis. Different lowercase letters were used to indicate significant differences. A one side Welch’s t-test method was used to compare differences between the two groups. The influence of patient phenotype on microbiome was determined using permanent multivariate analysis of variance (PERMANOVA) and redundancy analysis (RDA). The relationships between the microbiota and clinical factors were analyzed using the Spearman’s rank correlation test and the pheatmap package was used to draw a heatmap. * indicated a p <0.05 and ** indicated a p <0.01. The remaining visual images were created using ggplot2, the EasyMircoPlot package, or the MicrobiotaProcess package in R (Xu and Yu, 2022).

## 3 Results

### 3.1 Patient characteristics

The study participants were recruited from the First Affiliated Hospital of Jiamusi University. The healthy controls were recruited from the physical examination department and the patients were newly diagnosed with primary ITP. After strict screening, a total of 73 samples were collected, including 36 from the healthy control group and 37 cases from the patient group. The patients were in active disease at the time of blood sample collection. Fecal samples were collected at 4th week after the clinical treatment. The patient group was further subdivided into three treatment groups by medication type: 11 patients were in the Eltrombopag olamine treatment group (Eltro), 15 were in the Anzatax-leucogen group (Ami_Leu), and 11 were in the recombinant human interleukin-11 group (Leukin11). The platelet count (Pla) and platelet specific volume (PCT) of patients in the Ami_Leu group were significantly higher than those in the other two treatment groups. Other clinical indices, including age, sex, red blood cell count (RBC), hemoglobin (Hmb), and hematocytosis (Hem), were similar between all the three treatment groups (**Table 1**). PERMANOVA results showed no significant correlation between average patient age or the venereal diseases found in the disease group and the composition of the intestinal microbiota (P=0.372; P=0.291). Other clinical indices correlated significantly with the gut microbiota, with P values of 0.008, 0.022, 0.001, 0.049, 0.022 (**Supplementary Table S1**). Canonical correlation analysis (CCA) of the microbiota and clinical indices is shown in **Supplementary Figure S1**.

### 3.2 Sequencing characteristics

The samples were sequenced on illumina Novaseq6000 platform (Illumina, USA). The quality value 30 of the raw data was above 90% in each case. 2701824 readings were retrieved after filtering, and 2430825 tags were obtained after joining. Through repeated sampling and the elimination of low-frequency AVS, 10030 OTU sequences in total were acquired for downstream analysis. Detailed data of each sample were showed in **Supplementary Table S2**.

### 3.3 Composition and diversity of the gut microbiome in each group

**Figure 1:**
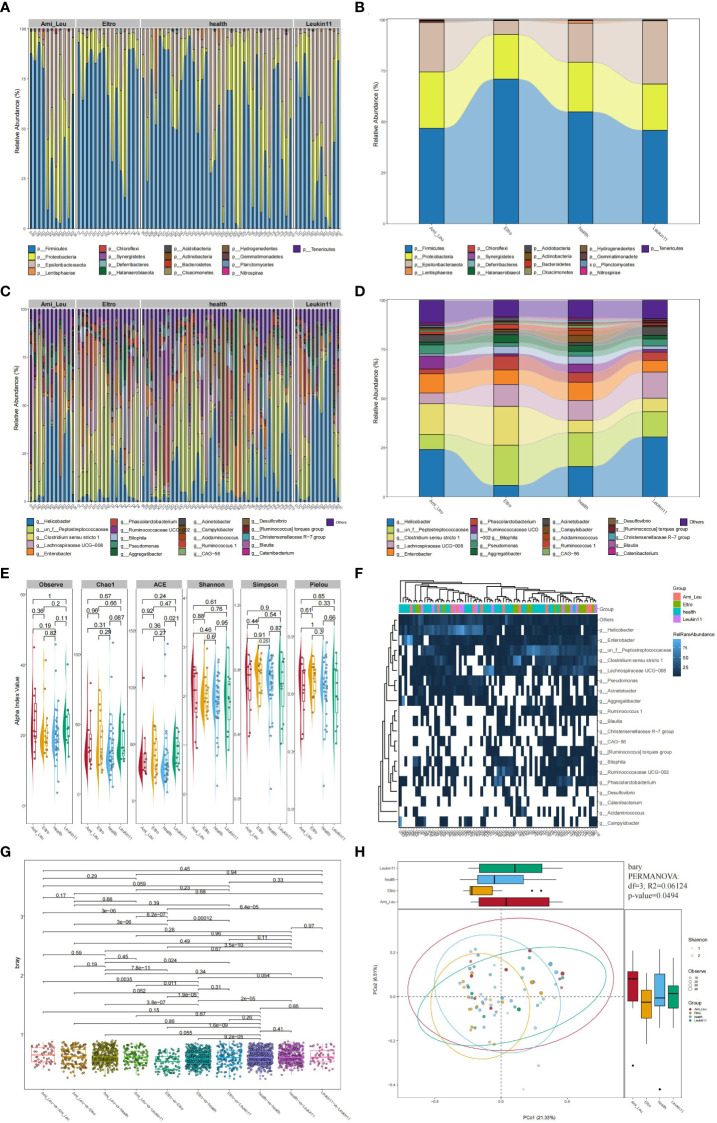
Composition of the gut microbiota in the healthy, Ami_Leu, Eltro, and Leukin11 groups. **(A–D)** The histogram and snaky diagram indicate the microbiome composition at the genus and phylum level. **(E)** Alpha diversity is shown using the Observe, Chao1, ACE, Shannon, Simpson, Pielou indices. **(F)** A heatmap of the top 20 microbiota at the genus level. **(G)** Comparison of the β diversity in samples from different groups using the unweighted UniFrac distance. **(H)** Principal coordinate analysis (PCoA) based on the unweighted UniFrac distance.

Bacteria with the highest abundance at the phylum classification level were *Firmicutes*, followed by *Proteobacteria* and *Epsilon bacteaeota*, which accounted for >90% of the gut microbiota. At the genus level, the top four bacteria were Helicobacter, Peptostreptococcaceae, Clostridium sensu stricto 1, and Lachnospiracea UCG-008, which accounted for >45% of the microbiome (**Figure 1A**). A Sankey diagram clearly showed differences in the proportion of bacteria at each microbial classification level (**Figures 1B–D**). Alpha diversity showed the difference between the treatment and the healthy groups (**Figure 1E**). A heatmap showed the abundance of the top 20 bacteria at the genus level (**Figure 1F**). PCoA and PERMANOVA analyses using the unweighted UniFrac distance revealed that the overall composition of the gut microbiota differed significantly among the four groups (R2 = 1.5004, P=0.0494) (**Figure 1H**). The difference between two groups is shown in **Figure 1G**.

### 3.4 Microbial co-occurrence networks of the different groups

**Figure 2:**
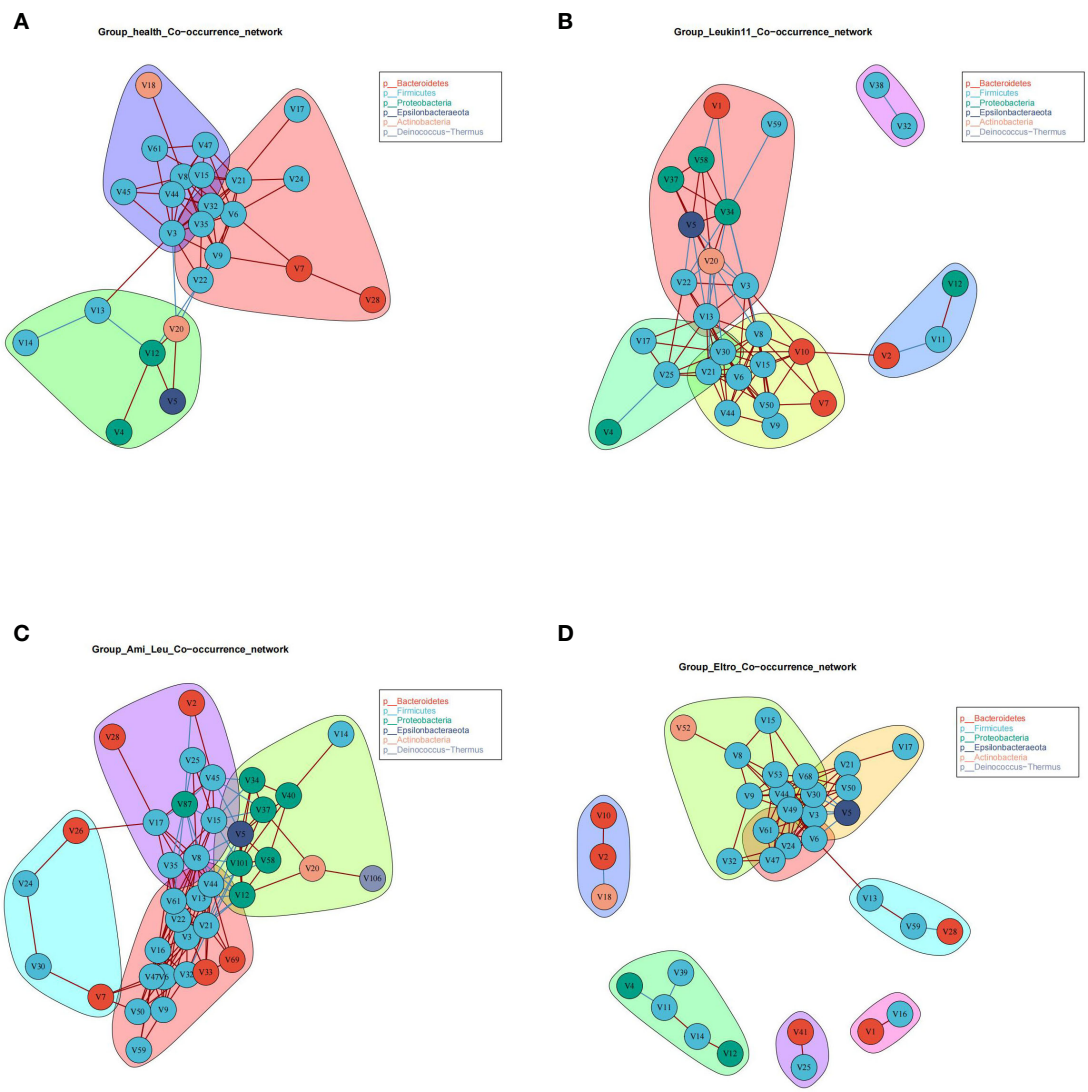
**(A–D)** Co-occurrence analysis of the gut microbiome in the four groups.

The healthy group and the three treatment subgroups had the same verticals but completely different cross talk (**Figure 2**). The core microbiota of the healthy group were primarily grouped into three clusters, indicating that the gut microbiota in this group had better synergistic effects. There were clear differences in the co-occurrence networks of the three treatment groups, indicating that drug treatment had a significant impact on the gut microbiota. Surprisingly, there was a V5 (*Helicobacter*) in the treatment groups, and the relative abundance of *Helicobacter* was significantly higher in the Ami_Leu and Leukin11 groups than in the healthy group. Given that *Helicobacter* is generally considered a pathogenic bacterium, it remains unclear why it was present and found in relatively high abundance.

### 3.5 Microbiome and random forest modeling

**Figure 3:**
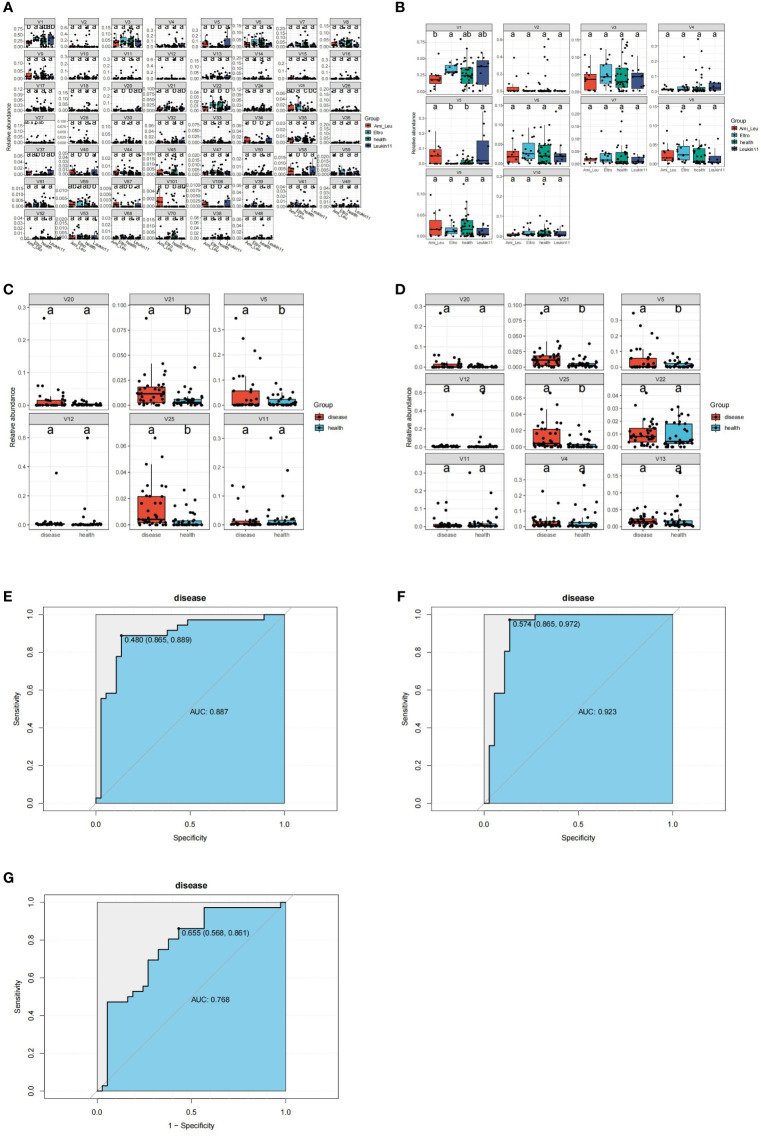
Identification of the signature gut microbiota by random forest. **(A)** Differential bacteria between the groups at the genus classification level. **(B)** Differential bacteria between groups at the phylum classification level. **(C)** Differential bacteria between health group and disease group at the phylum classification level. **(D)** Differential bacteria between health group and disease group at the genus classification level. **(E, F)** Area Under the Curve (AUC) showing the diagnostic accuracy of the intersect and union gut microbiota. **(G)** AUC showing the diagnostic accuracy of V5 (g:Helicobacter), V21(g:Ruminococcus 1), and V25(g:Clostridium sensu stricto 1).

Gut microbiota are often used as biomarkers to assess differences between groups. In this study the tax_diff function of Easymicroplot software was used to calculate differences in the bacteria in each group. Bacteria in the three treatment groups and the healthy group differed at the phylum and genus levels (**Figures 3A, B**). At the phylum level, four bacteria, V4 (*Actinobacteria*), V5 (*Epsilonbacteraeota*), V10 (*Epsilonbacteraeota*), and V11 (*Deinococcus Thermus*) differed between the groups. At the genus level, 15 bacteria differed significantly between the treatment and healthy groups. The abundance of all the significantly altered microbiota was high in the treatment groups. Random forest modeling was used to cross validate and explore biomarkers to distinguish between the healthy and treatment groups at phylum or genus level (**Figures 3C, D**). The key microbiome of intersection and union was better at distinguishing between the control and treatment groups (see key microbiome detail in **Supplementary Table 3**) (**Figures 3E, F**). The results of the ROC analysis for the key bacteria V5 (*Helicobacter*), V21 (*Ruminococcus 1*), and V25 (*Lachnospiraceae NK4A136 group*) revealed that the value of AUC was 0.768, which could essentially distinguish the two groups, but it was still subpar compared to the outcome of the random forest modeling (**Figure 3G**).

### 3.6 Differences in the microbial function of each group

**Figure 4:**
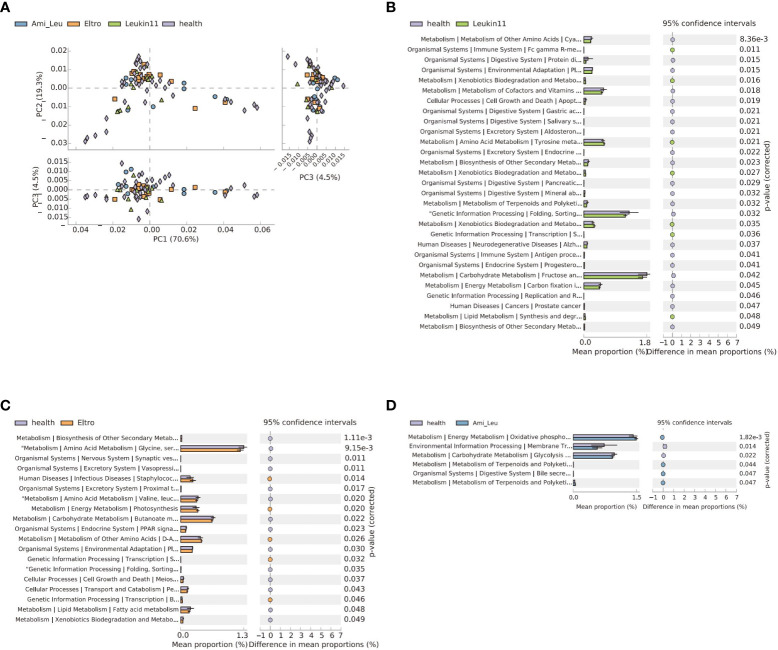
The gut microbiota functional changes in different groups. **(A)** Principal Component Analysis of pathways in the four groups. **(B–D)** Extended error bar of the three treatment groups versus the healthy group. The p-value indicates a significant difference between the two groups.

The microbial function differed between the treatment groups and the healthy group. The Leukin11 and the healthy groups had the largest difference in function, followed by the Eltro and Ami_ Leu groups, which could be related to differences between the therapeutic drugs. Analysis of the different treatment subgroups and the healthy group showed that 60% of the top 10 pathways were related to compound synthesis, three of which were related to terpenoid metabolism (**Figures 4A–D**). Detailed results of the differences between the groups is shown in **Supplementary Table S3** and **Figure S2**.

### 3.7 Identification of the relationship between clinical indices and significant microbiota

**Figure 5:**
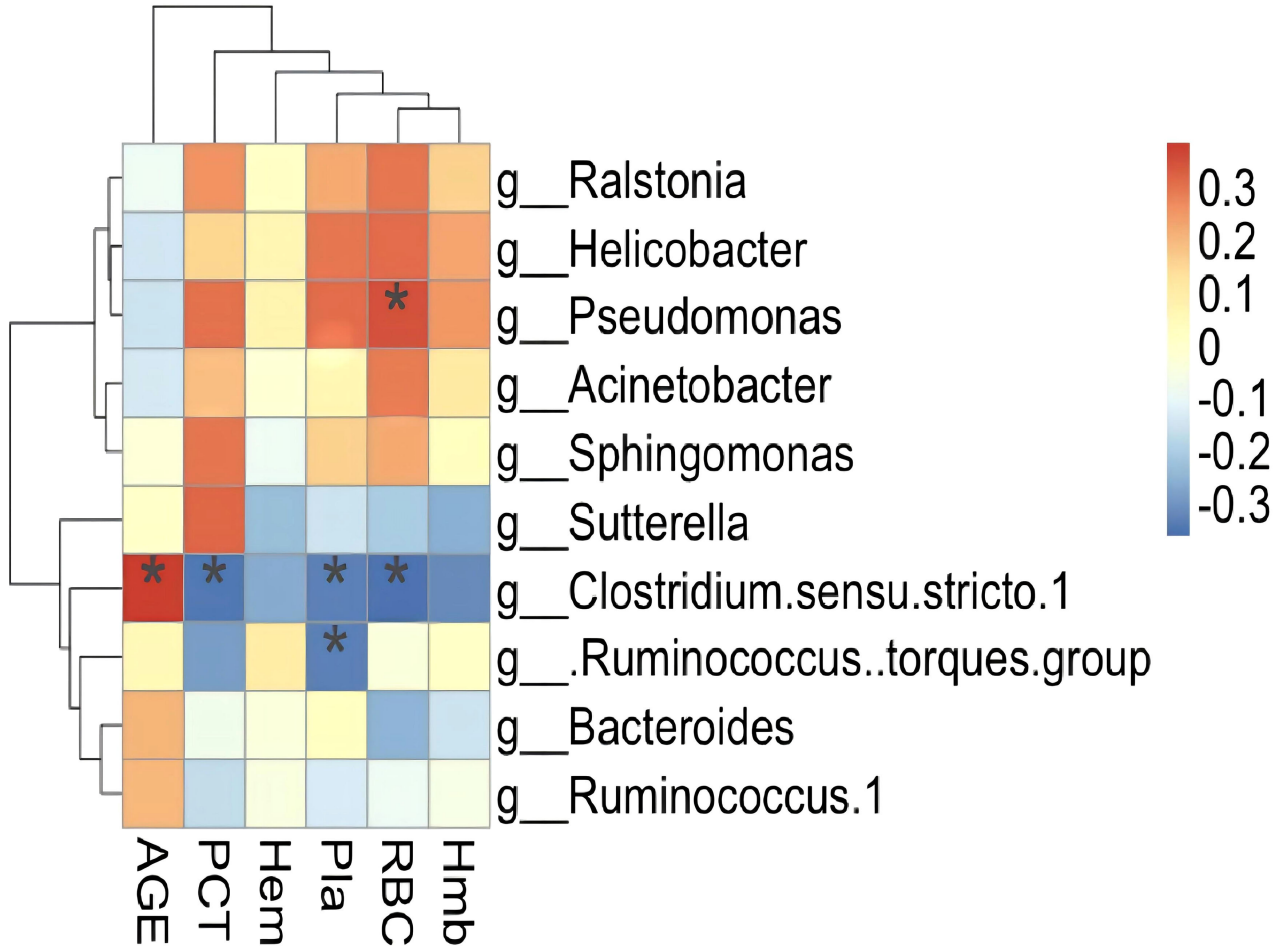
Heatmap showing significant correlations between the gut microbiota and the libratory test. AGE, age of patients; RBC, red blood cell; Hmb, hemoglobin; Hem, Hematocytosis; Pla, Platelet count; PCT, Thrombocytosis. Notes on the right represent microbial classification at the genus level; *P <0.05.

Spearman’s correlation analysis was used to investigate the relationship between clinical indices and significant deferential microbiota and a heat-map was created to show the results. Ten differential microbiota clustered into two groups, one positive and one negative. The negatively correlated bacteria were all members of *Clostridiales*, while the positively correlated bacteria were mainly pathogens and opportunistic pathogens. Three bacteria correlated significantly with clinical indicators, two of which correlated negatively with platelet count (**Figure 5**).

## 4 Discussion

16s rRNA sequencing is a mature technology that is widely used to study the interaction between intestinal microorganisms and host immunity. To date, researchers have identified more than 20 autoimmune diseases related to changes in the gut microbiome, including type I and II diabetes, multiple sclerosis, rheumatoid arthritis, and inflammatory bowel disease (Qin et al., 2012; Jie et al., 2017; Wen et al., 2017; Collison, 2019; Franzosa et al., 2019). However, metagenome sequencing technology has rarely been used to study ITP. The current study compared the microbial composition and function of the intestinal microbiome of ITP patients in different drug treatment groups and healthy controls, and determined the correlation between clinical indices and differential microbiota using 16s rRNA sequencing. The findings indicated that at the phylum level, *Proteobateria*, *Epsilon bactriaeota*, *Bacteroidetes*, and *Firmicutes* were significantly increased in the different treatment groups. The significantly increased bacteria at the genus level were also members of these phyla in the treatment groups. Random forest modeling analysis showed that these key bacteria had the ability to distinguish between the treatment and healthy control groups, a finding validated by the ROC curve (union AUC value:0.923, intersect AUC value:0.887) (**Figures 3E, F**). These results indicate that the key bacteria have diagnostic value. Pathways that differed between the treatment and the healthy groups primarily related to the terpenoid and polyketide metabolism and biosynthesis of other secondary metals. This may be due to changes that occur in the intestinal microbiome in response to drug treatment.

The ratio of *Bacteroidetes* to *Ferricules* serves as a measure of an imbalanced gut microbiome. For example, in obese people, *Bacteroidetes/Ferricules* ratio decreases, while in SLE patients, the ratio increases (Hevia et al., 2014; Liu et al., 2017). The current study did not observe significant changes in the *Bacteroidetes/Ferricules* ratio, mainly because the relative abundance of *Firmicutes* was half of the total microbiome, while the proportion of *Bacteroidetes* was relatively low. However, it was observed that 50% of the bacteria with significant differences were members of *Proteobateria*, and the relative abundance of *Proteobateria* was significantly higher in the treatment groups than in the healthy group. Innate immune response disorders are shown to promote the growth of *Proteobacteria* in mice. Animals with an IL-10 deficiency develop spontaneous colitis due to their inability to tolerate the intestinal microbiome (Maharshak et al., 2013). In addition, the relative abundance of *Ruminococcus 1* increases in rats with polycystic ovary syndrome after treatment with tempol, which may help to reduce oxidative stress and restore the intestinal ecology (Li et al., 2021). In the current study, the relative abundance of *Clostridium sensu stricto 1* was significantly higher in the treatment groups than in the healthy group. *Clostridium sensu stricto 1*, a beneficial microorganism that produces butyric acid, together with *Ruminococcus 1*, maintains the intestinal barrier and plays an important role in regulating immune and anti-inflammatory processes (Wen et al., 2021). Surprisingly, while we excluded sample and sequence processing data contamination, the relative abundance of *Helicobacter* was still higher in the treatment groups (except the Eltro group) than in the healthy control group. Prior research has shown that patients with *Helicobacter pylori* infection may develop ITP. Platelet counts return to normal in approximately 50% of patients after *Helicobacter pylori* eradication, making this a viable option for ITP treatment (Veneri et al., 2005). This was a deficiency in this study, which showed Helicobacter pylori to be present in the treatment group. Short-chain fatty acids, for example, encourage intestinal wall cells to breathe oxygen by activating beta oxidation, stimulating intestinal wall cells to do so in order to allow anaerobic pathogens to proliferate and safeguard the host’s intestinal health (Zafar and Saier, 2021). Drugs, such as Tempol, can promote the proliferation of intestinal microorganisms through oxidative stress. They can also be used to improve the glucose metabolism pathway by metabolizing bile acid and other products, maintaining host immunity and homeostasis (Li et al., 2021). The current study showed that *Bacteroidetes* and *Firmicutes* were significantly increased in the treatment groups. These bacteria may regulate intestinal wall cell activity and enhance host immune function by producing acetic acid, butyric acid and other short chain fatty acids. Functional analysis of the gut microbiome revealed that the difference in KEGG level 3 between the treatment and healthy groups was mainly in terpenoid and polyketone metabolism, peripheral biodegradation and metabolism, and amino acid metabolism. Terpenoids and polyketones are shown to have anti-tumor activity (Huang et al., 2012; Gomes et al., 2013). These pathways were enriched in the treatment groups, indicating that treatment improves the intestinal microenvironment, promotes the biosynthesis of secondary metabolites and increases the host immune state.

This study assessed patients receiving one of three different treatments for ITP, Etripopal, recombinant human interleukin 11 for injection, and aminopeptide and risperidone. After treatment, the abundance of *Proteobacteria*, *Epsilon bacteraeota*, *Bacteroidetes* and *Firmicutes* were significantly higher in the intestinal microbiome. Correlation analysis of the blood indices and the differential bacteria also revealed a negative correlation between the platelet count and several Firmicutes and Bacteroides species, but a positive correlation with diseases like *Helicobacter* and *Pseudomonas*. It may be considered to treat ITP patients by enhancing the aforementioned bacteria, which may be a novel clinical approach since random forest modeling research demonstrated that the main distinct microbiota can well identify the healthy group and the treatment group.

The study also had the following limitations. Although the data showed microbial differences between treatment groups and the healthy control group and identified certain critical microorganisms associated with medication treatment and clinical benefits. First, we should note that changes in intestinal bacteria are also influenced by the host’s diet, alcohol use, level of stress, and other factors; these phenotypic data have not been gathered. Second, animal tests to confirm the relationship between distinct microbial metabolites and platelet count were not included in this study. Third, because this was a single-center study with a small number of specimens obtained, the results need to be confirmed by data from many centers.

## 5 Conclusion

In summary, our study provided insights into alterations of the gut microbiota between the treatment group and the healthy group. Specifically, at the level of genus classification, the majority of bacterial in the healthy group exhibited higher abundance than those in the treatment group, these altered microbiota have great potential for the diagnosis of IPT diseases. Moreover our findings suggest that Pseudomonas was significantly enriched in the treatment group, this bacterial showed positive correlation of platelet count. This observation indicates a potentially important role of Pseudomonas in the pathogenesis and treatment mechanism of IPT. Identifying key microbiota in gut microbiota and elucidating their functions represents a promising avenue for future IPT disease treatment.

## Data availability statement

The datasets presented in this study can be found in online repositories. The names of the repository/repositories and accession number(s) can be found below: https://db.cngb.org/search/?q=CNP0004135.

## Ethics statement

The studies involving human participants were reviewed and approved by the ethics committee of Nanjing Red Cross Blood Center. The patients/participants provided their written informed consent to participate in this study.

## Author contributions

XR and YF performed the experiments, analyzed the data and wrote the manuscript. XR and YF made the equal contribution to the article. CH and QF contributed to the research design and modified the manuscript. YZ and JC provided technical support. All authors contributed to the article and approved the submitted version.

## Supplementary material

**Table S1** showed the summary table of sequencing data. **Table S2** and **Figure S1** showed the results of PERMANOVA and canonical correlation analysis of the microbiota and clinical indices. **Table S3** and **Figure S2** showed the function of difference in different groups. **Table S4–S6** showed the correspondence between letters and microorganisms.
