# Supplementary figures and images for: Gut microbiota were altered with platelet count and red blood cell count in immune thrombocytopenia patients with different treatments
Source: Front Cell Infect Microbiol. 2023 May 15;13:1168756. doi: 10.3389/fcimb.2023.1168756 (PMC10225573; doi:10.3389/fcimb.2023.1168756)

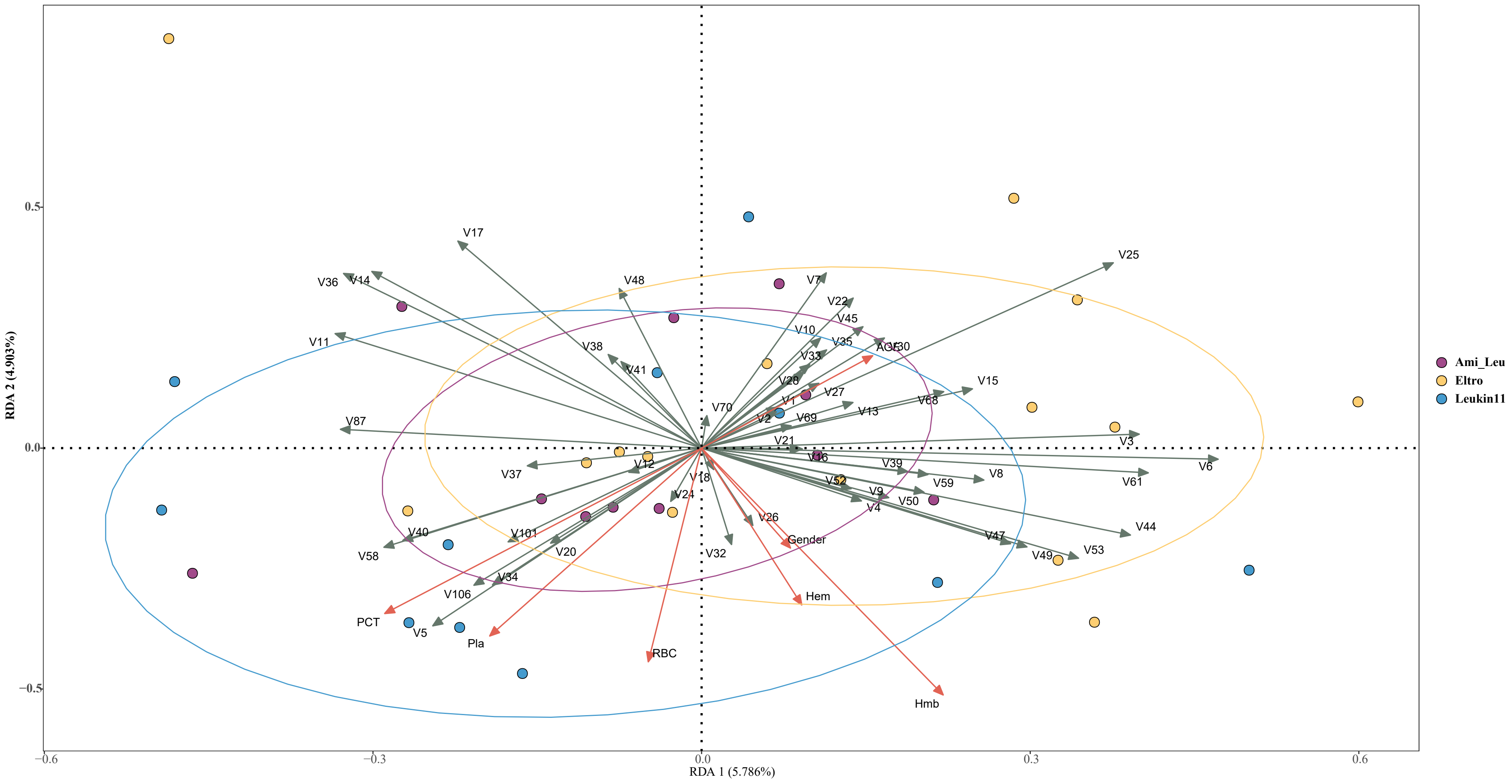

Supplement: Supplementary file 1 [file DataSheet_1.pdf]

A

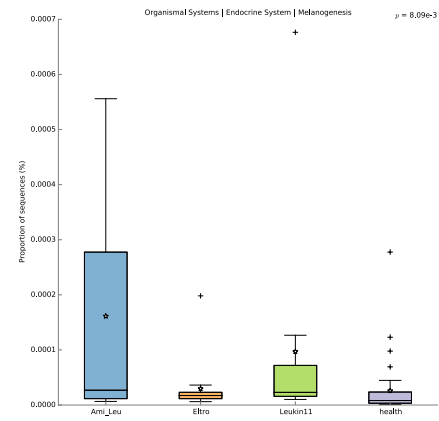

B

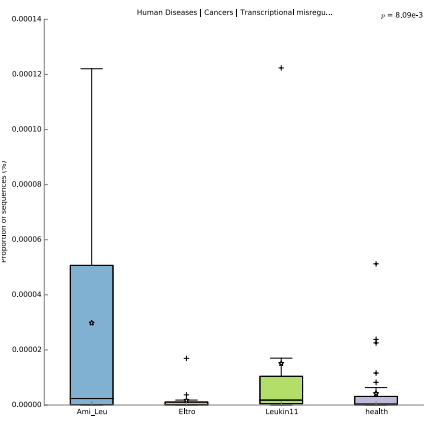

C

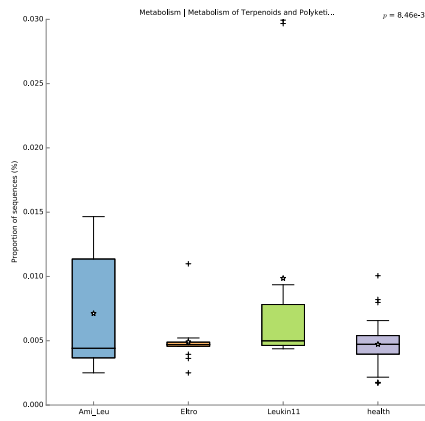

D

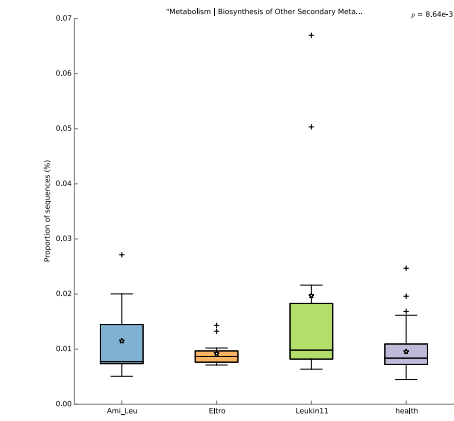

E

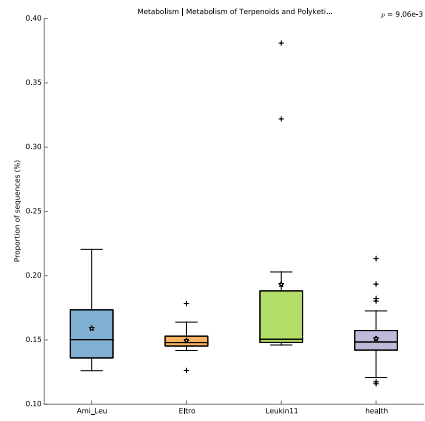

F

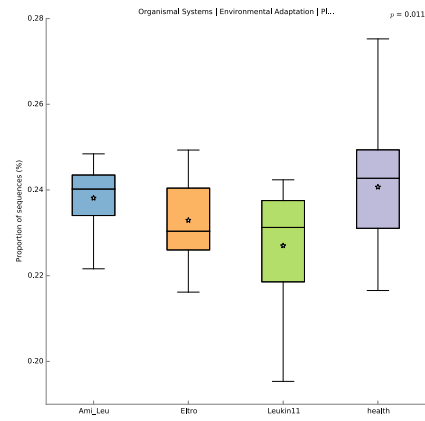

G

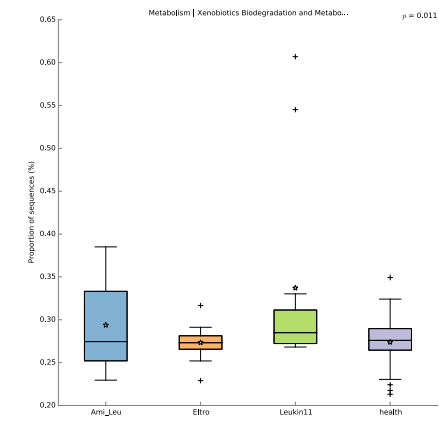

H

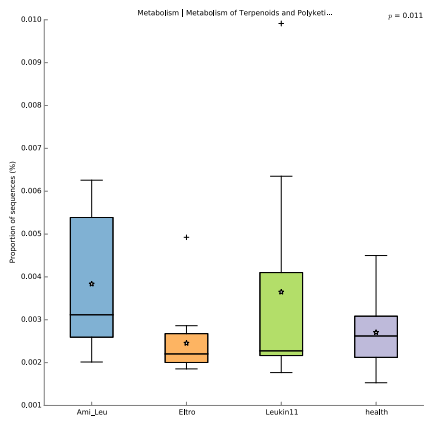

I

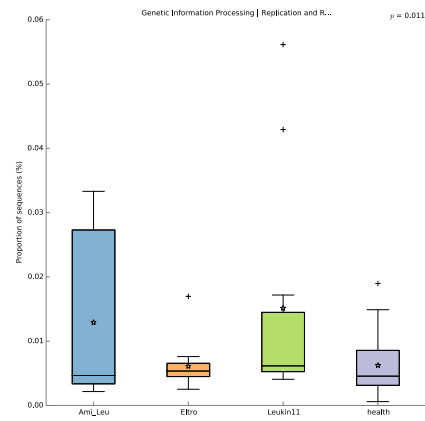

J

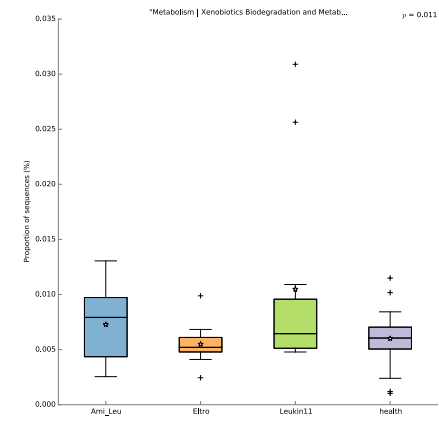

Supplement: Supplementary file 8 [file DataSheet_8.pdf]

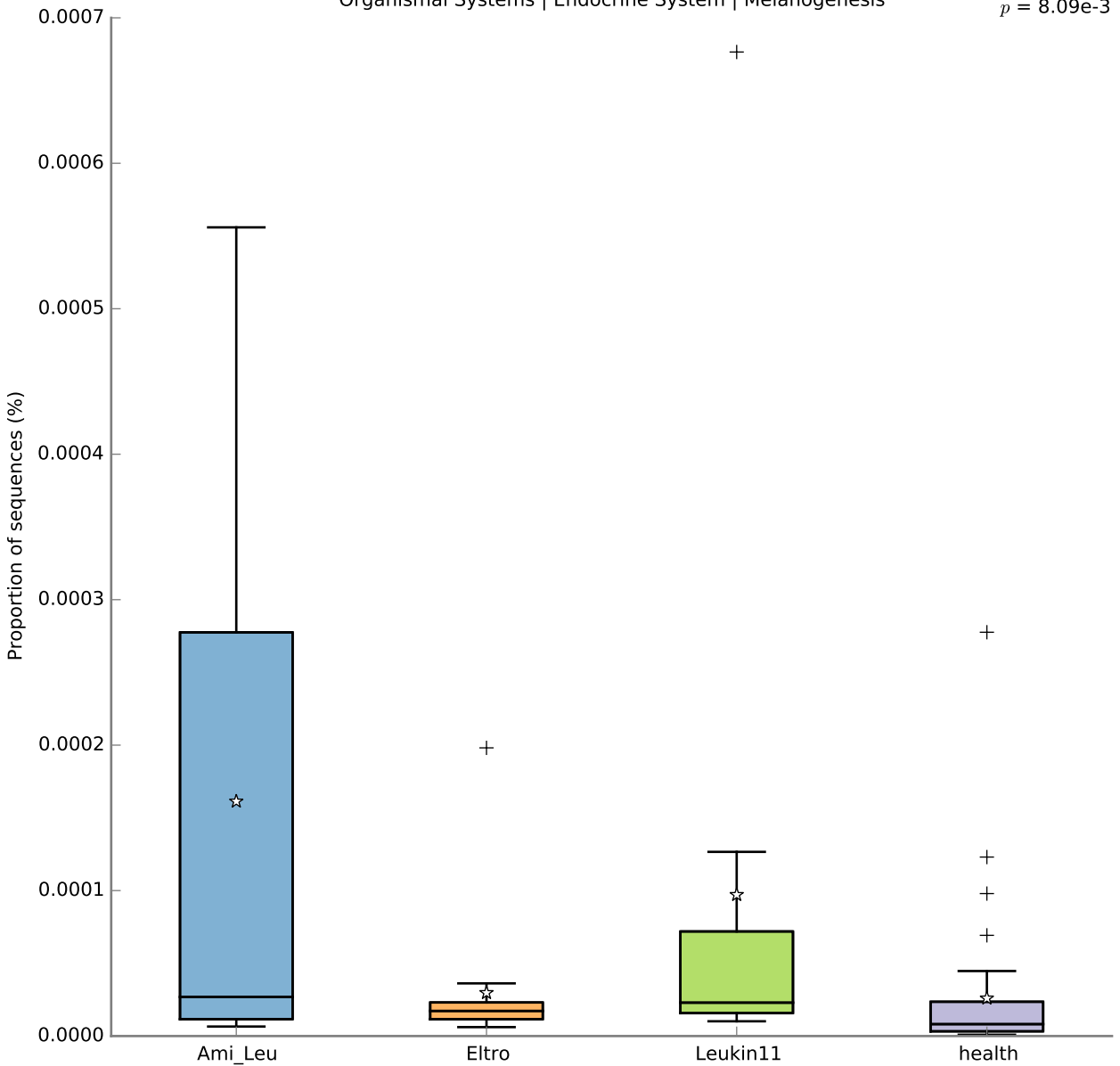

Supplement: Supplementary file 9 [file DataSheet_9.pdf]

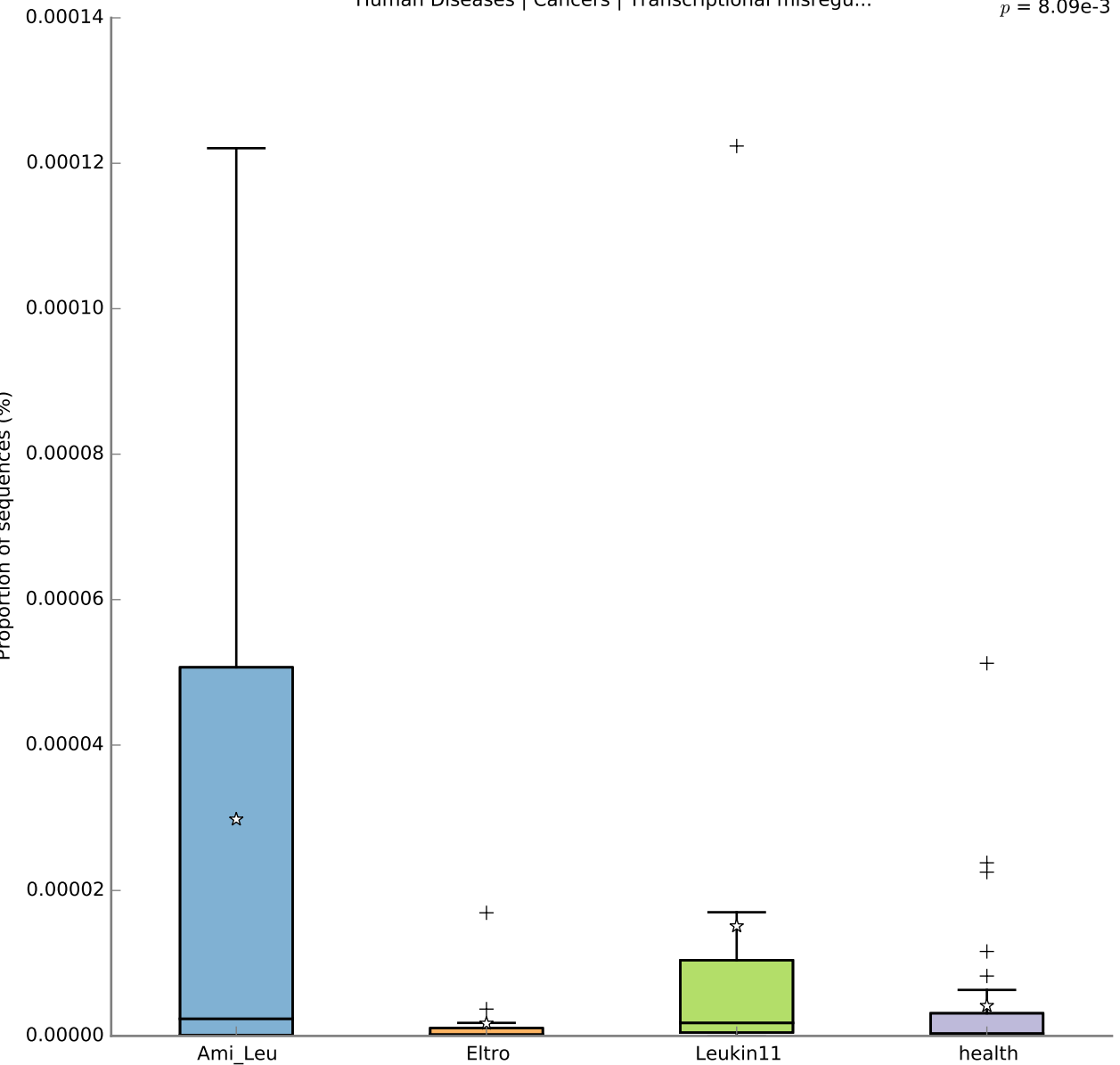

Supplement: Supplementary file 10 [file DataSheet_10.pdf]

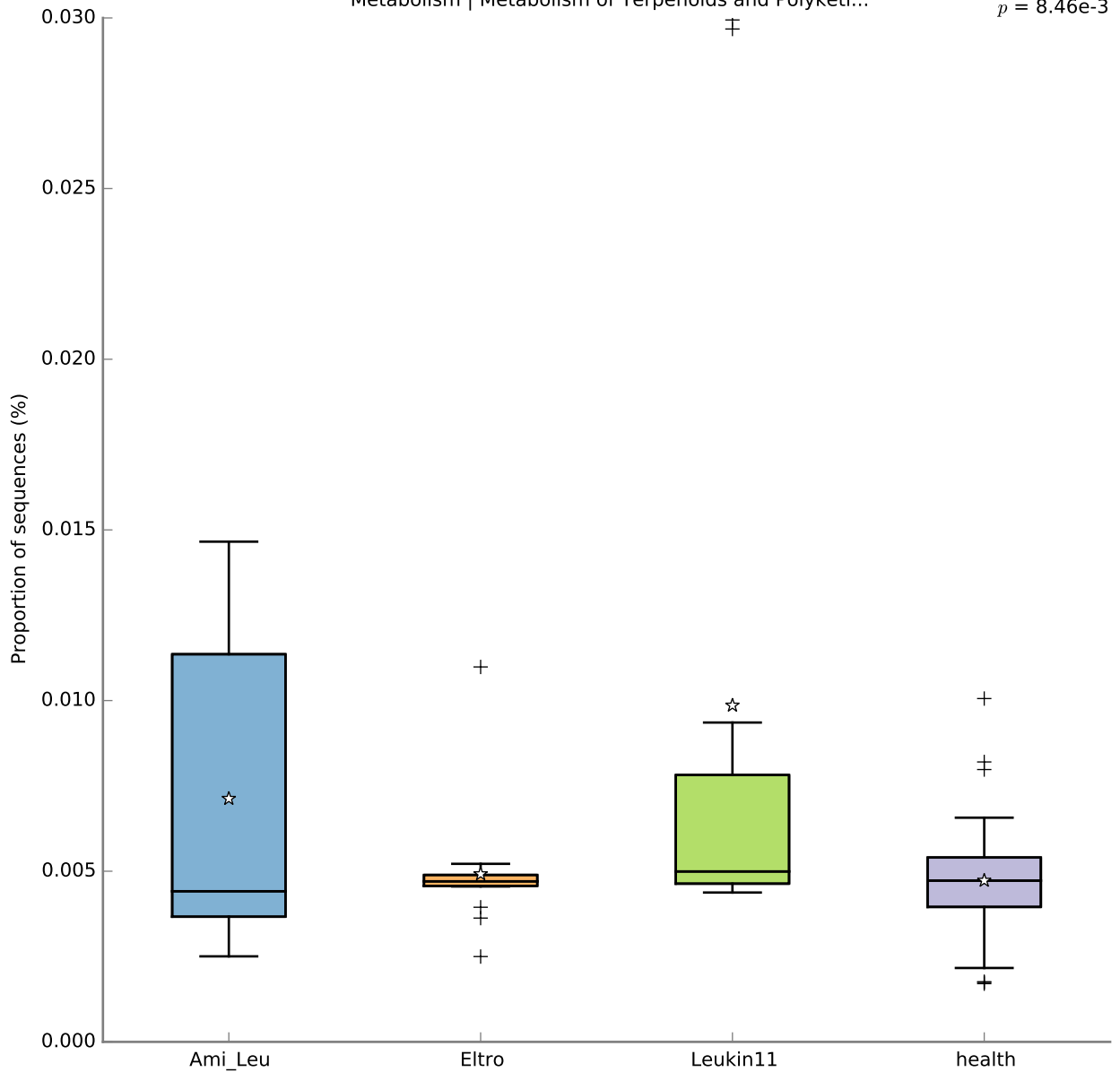

Supplement: Supplementary file 11 [file DataSheet_11.pdf]

"Metabolism | Biosynthesis of Other Secondary Meta...

$p = 8.64\text{e-}3$

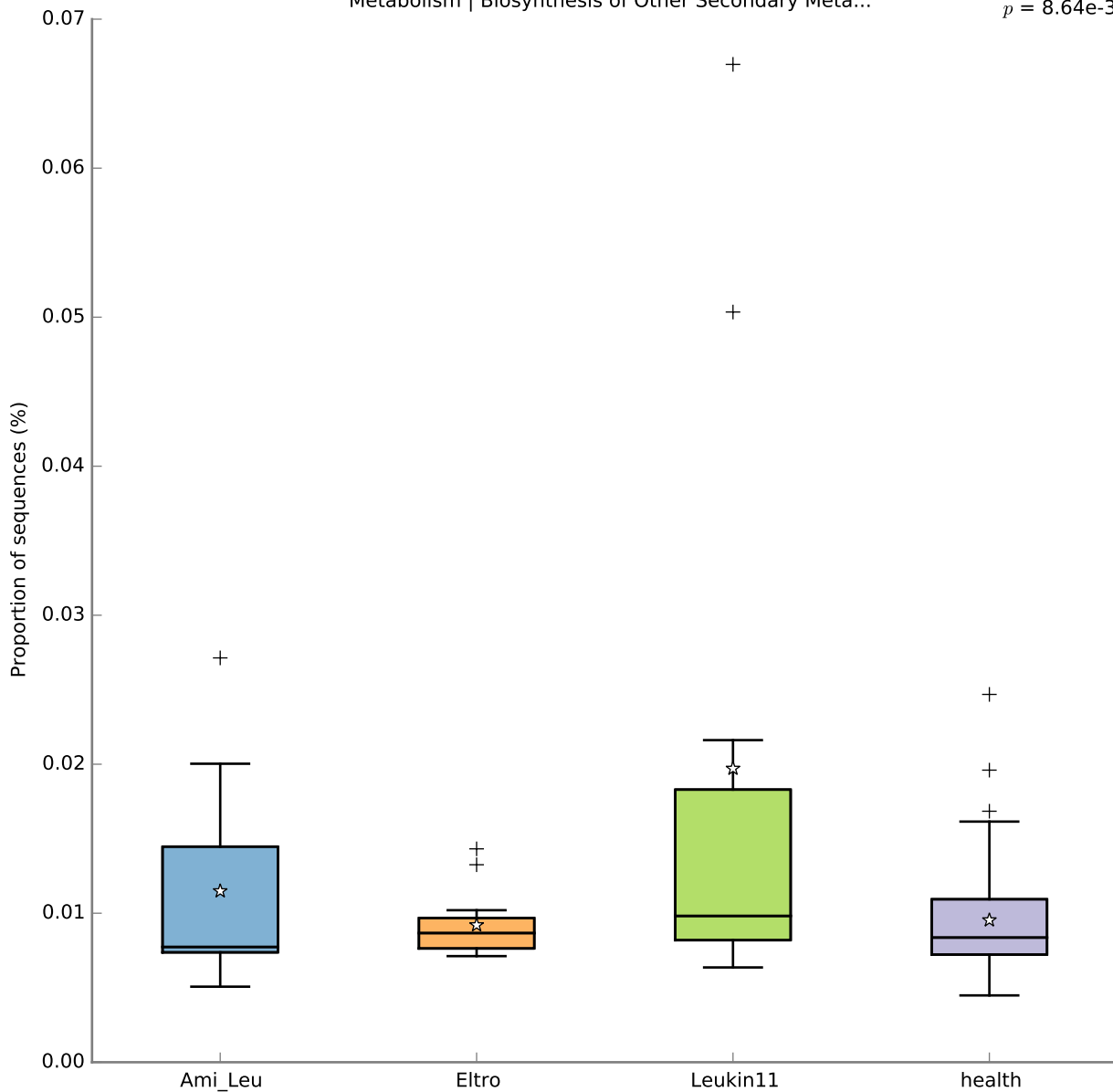

Supplement: Supplementary file 12 [file DataSheet_12.pdf]

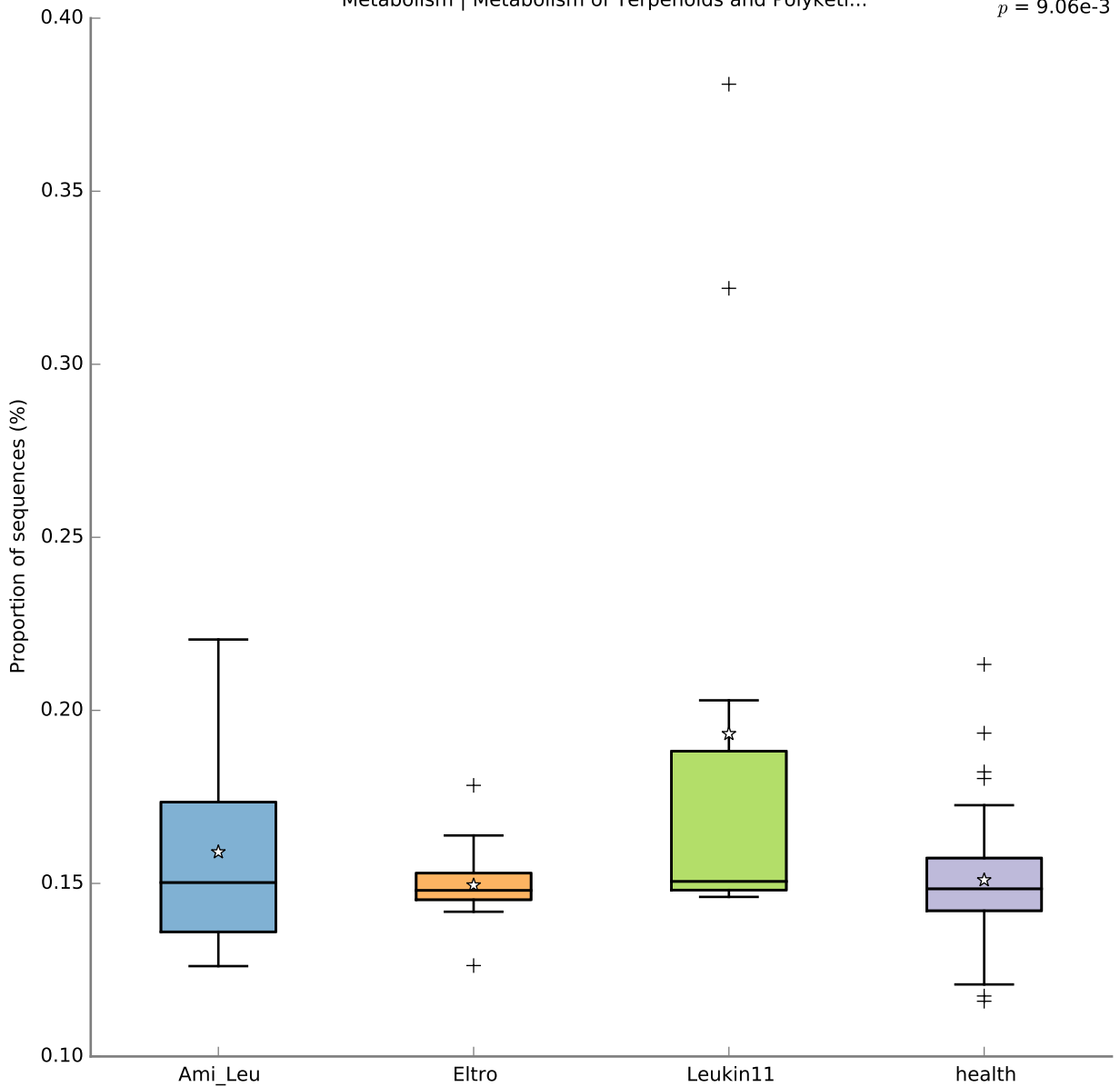

Supplement: Supplementary file 13 [file DataSheet_13.pdf]

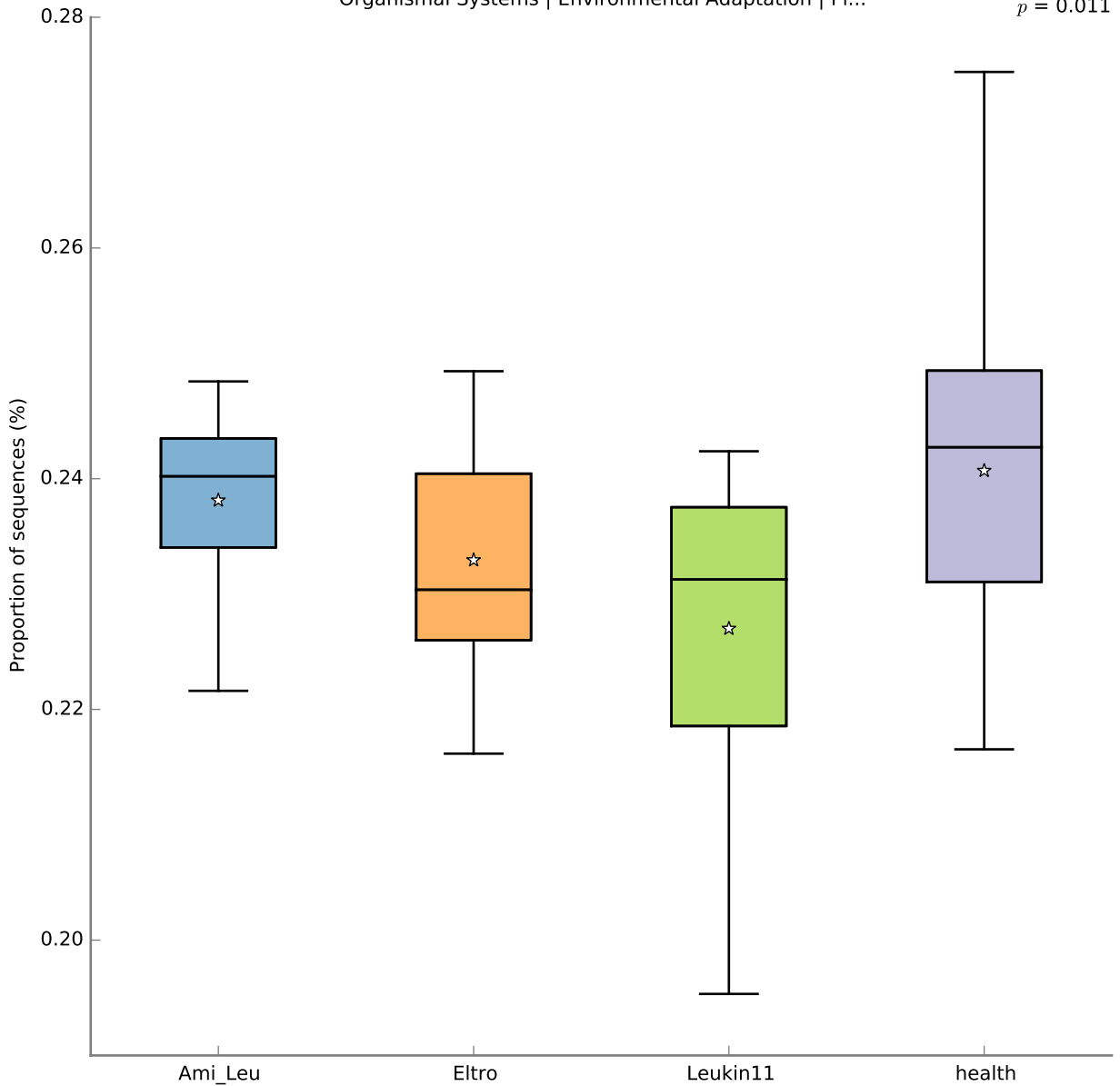

Supplement: Supplementary file 14 [file DataSheet_14.pdf]

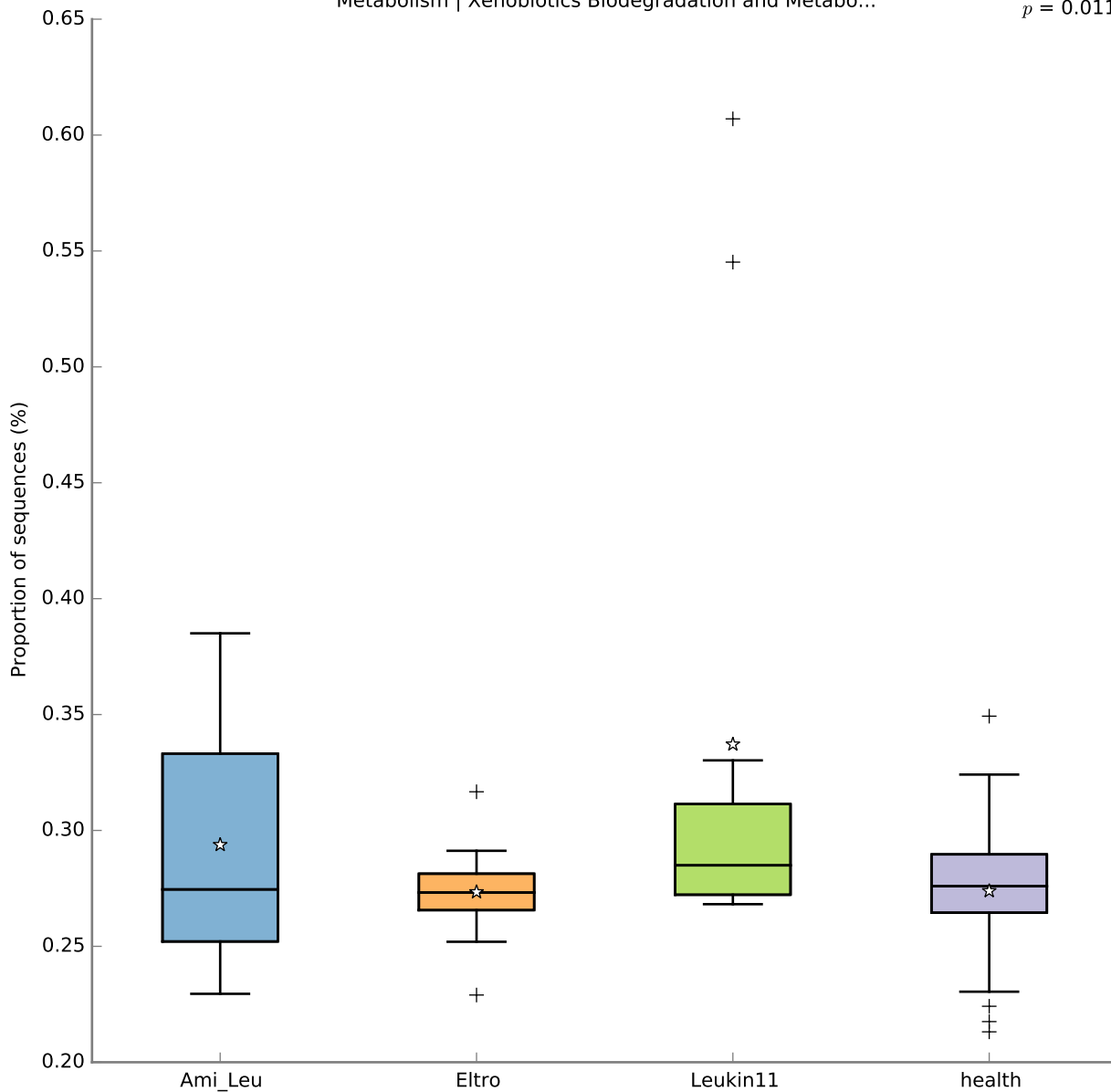

Supplement: Supplementary file 15 [file DataSheet_15.pdf]
